# Long-term clinical outcomes of patients with COVID-19 and chronic liver disease: US multicenter COLD study

**DOI:** 10.1097/01.HC9.0000897224.68874.de

**Published:** 2023-01-03

**Authors:** Elizabeth S. Aby, Ghady Moafa, Nyan Latt, Mohammad T. Sultan, Paula A. Cacioppo, Sonal Kumar, Raymond T. Chung, Patricia P. Bloom, Jenna Gustafson, Michael Daidone, Zoe Reinus, Jose D. Debes, Sunny Sandhu, Aalam Sohal, Sameeha Khalid, Marina Roytman, Andreea Maria Catana, Kara Wegermann, Rotonya M. Carr, Yedidya Saiman, Ihab Kassab, Vincent L. Chen, Atoosa Rabiee, Carly Rosenberg, Veronica Nguyen, Christina Gainey, Kali Zhou, Kenneth Chavin, Blanca C. Lizaola-Mayo, David M. Chascsa, Lee Varelas, Akshata Moghe, Renumathy Dhanasekaran

**Affiliations:** 1 Hennepin County Medical Center, Minneapolis, Minnesota, USA; 2 University of Minnesota, Minneapolis, Minnesota, USA; 3 Ochsner Medical Center, New Orleans, Louisiana, USA; 4 Weill Cornell Medical Center, New York, New York, USA; 5 Massachusetts General Hospital, Boston, Massachusetts, USA; 6 University of California San Francisco, Fresno, California, USA; 7 Beth Israel Deaconess Medical Center, Boston, Massachusetts, USA; 8 Duke University, Durham, North Carolina, USA; 9 University of Pennsylvania, Philadelphia, Pennsylvania, USA; 10 University of Michigan, Ann Arbor, Michigan, USA; 11 VA Medical Center, Washington, DC USA; 12 University of Arizona/BannerHealth, Tucson, Arizona, USA; 13 University of Southern California, Los Angeles, California, USA; 14 University Hospitals Cleveland Medical Center, Cleveland, Ohio, USA; 15 Mayo Clinic, Scottsdale, Arizona, USA; 16 University of Pittsburgh, Pittsburgh, Pennsylvania, USA; 17 Stanford University, Stanford, California, USA

## Abstract

COVID-19 is associated with higher morbidity and mortality in patients with chronic liver diseases (CLDs). However, our understanding of the long-term outcomes of COVID-19 in patients with CLD is limited.

**Methods:**

We conducted a multicenter, observational cohort study of adult patients with CLD who were diagnosed with COVID-19 before May 30, 2020, to determine long-term clinical outcomes. We used a control group of patients with CLD confirmed negative for COVID-19.

**Results:**

We followed 666 patients with CLD (median age 58 years, 52.8% male) for a median of 384 (interquartile range: 31–462) days. The long-term mortality was 8.1%; with 3.6% experiencing delayed COVID-19-related mortality. Compared to a propensity-matched control group of patients with CLD without COVID-19 (n=1332), patients with CLD with COVID-19 had worse long-term survival [*p*<0.001; hazards ratio (HR): 1.69; 95% CI: 1.19–2.41] and higher rate of hospitalization (*p*<0.001, HR: 2.00, 1.62–2.48) over a 1-year follow-up period. Overall, 29.9% of patients reported symptoms of long-COVID-19. On multivariable analysis, female sex (*p*=0.05, HR: 2.45, 1.01–2.11), Hispanic ethnicity (*p*=0.003, HR: 1.94, 1.26–2.99), and severe COVID-19 requiring mechanical ventilation (*p*=0.028, HR: 1.74, 1.06–2.86) predicted long-COVID-19. In survivors, liver-related laboratory parameters showed significant improvement after COVID-19 resolution. COVID-19 vaccine status was available for 72% (n=470) of patients with CLD and history of COVID-19, of whom, 70% (n=326) had received the COVID-19 vaccine.

**Conclusions:**

Our large, longitudinal, multicenter study demonstrates a high burden of long-term mortality and morbidity in patients with CLD and COVID-19. Symptoms consistent with long-COVID-19 were present in 30% of patients with CLD. These results illustrate the prolonged implications of COVID-19 both for recovering patients and for health care systems.

## INTRODUCTION

The COVID-19 pandemic, first reported in late 2019, continues to have a devastating effect on global health with significant associated morbidity and mortality.^1^ While the clinical characteristics and complications of acute COVID-19 have been well described,^2^ long-term outcomes after recovery from COVID-19, particularly related to individuals with liver disease, remain unknown. Early studies raised concern that patients who survived at least 30 days from the diagnosis of COVID-19 remain at higher long-term risk of death.^3^,^4^ Evolving scientific evidence also suggests that those infected with COVID-19 can experience long-lasting debilitating symptoms, even months after being infected with COVID-19, termed “long-COVID-19.”^5^,^6^ The term long-COVID-19 encompasses the associated physical, medical, and cognitive sequelae post-COVID-19 infection with a reported prevalence between 30% and 54%.^7^ The prevalence of these long-term sequelae of COVID-19 in patients with chronic liver disease (CLD) is not known.

Our group conducted a multicenter observational cohort study of patients with CLD who acquired COVID-19 early during the pandemic (NCT04439084), and identified risk factors for early morbidity and mortality from COVID-19 in patients with CLD.^8^–^11^ We have now performed longitudinal follow-up of this patient cohort to understand the long-term outcomes of COVID-19 in patients with CLD. We sought to determine if patients with CLD and COVID-19 had a higher long-term mortality and morbidity after initial survival beyond 30 days. Patients with CLD who were confirmed to be negative for SARS-CoV-2 were used as a control group. We also evaluated the prevalence of long-COVID-19 and identified the risk factors for long-COVID-19 in patients with CLD. As more and more patients recover from COVID-19, it will be critical to decipher their long-term clinical course. Our study sheds light on the natural history of COVID-19 sequelae in patients with CLD and underscores their need for ongoing multidisciplinary management.

## METHODS

### Study design

The consortium of investigators to study COVID-19 in chronic liver disease (COLD) study was formed on April 14, 2020 (registered Clinicaltrials.gov NCT04439084). A total of 17 centers in the US participated in this multicenter observational cohort study (Supplemental Table 1, http://links.lww.com/HC9/A0). The institutional review board of each participating center reviewed and approved the study protocol. Adults (age above 18 y) with RT PCR-confirmed diagnosis of COVID-19 between March 1 and May 30, 2020, and preexisting CLD [according to predefined International Classification of Diseases-10 (ICD-10) codes listed and confirmed by manual chart review, listed in Supplemental Table 2, http://links.lww.com/HC9/A0] were included. AASLD guidelines were used to determine the etiology of the CLD. Patients were determined to have CLD related to hepatitis C if they had a prior history of hepatitis C and underwent treatment with sustained virologic response or if they had positive hepatitis C RNA on PCR-based testing. Patients were determined to have CLD related to hepatitis B if their hepatitis B surface antigen was positive. Diagnosis of cirrhosis was confirmed by documentation of fibrosis either by magnetic resonance elastography, fibroscan, FIB-4, or biopsy. Diagnosis of cirrhosis was ascertained in other patients by detailed chart review for clinical, radiological, or biochemical evidence of liver cirrhosis. Data on decompensation was collected from chart review for clinical events. Presence and severity of ascites, encephalopathy, variceal bleeding, and other major decompensating events were collected. The presence of comorbid conditions, such as diabetes and hypertension, were determined by ICD-10 codes along with manual chart review.

All the participating institutions systematically performed a review of their electronic medical records to identify all patients meeting the inclusion criteria. Further details on study definitions have been previously published.^8^,^9^

All participating institutions independently identified patients meeting inclusion criteria by searching their electronic medical records and collected data on clinical outcomes, long-COVID-19 symptoms, laboratory tests, vaccination status, alcohol use, weight changes, as well as other health and behavioral factors. All data were collected until death or date of last follow-up. Death was attributed to COVID-19 if it was clinically related to COVID-19 illness, and there were no other unrelated causes of death, as per the CDC guidelines.^12^ Liver injury was defined as alanine transaminase (ALT) values >2 times upper limit of normal with the cutoffs for normal values being 19 U/L for women and 30 U/L for men.^9^,^13^ The World Health Organization (WHO) definition was used to determine long-COVID-19.^14^ Long-COVID-19 was therefore defined as persistent symptoms which began at the time of COVID-19 diagnosis and lasted for more than 3 months in the absence of another clear explanation. The investigators in the study performed a detailed chart review to collect all the details regarding the symptoms of long-COVID-19 and also performed a detailed evaluation for alternate explanation for the symptoms. The definition of long-COVID-19 in published studies varies greatly, however, symptom duration of 3 months is the most prevalent definition of long-COVID-19 across studies.^7^ The National Institute on Alcohol Abuse and Alcoholism definition of alcohol use was used to define moderate, heavy, and binge drinking.^15^

### Control group with CLD without COVID-19

We collected data from consecutive patients with CLD at a single center (Stanford University) who did not have COVID-19 and tested negative for COVID-19 during the same period. We further confirmed these patients never tested positive for SARS-CoV-2 throughout the entire follow-up period. The same ICD codes used to identify patients with CLD for the COLD study were used to identify patients for the control group (Supplemental Table 2, http://links.lww.com/HC9/A0). We only included patients who had a minimum follow-up of 30 days.

### Statistical analysis

A predefined statistical data analysis plan was followed. Continuous variables are expressed as medians and interquartile ranges (IQRs) or mean and SD, as appropriate. Categorical variables are summarized as counts and percentages. The statistical significance of differences between groups was evaluated by using the independent *t* test or the Mann-Whitney *U* test for continuous variables and the χ^2^ test for categorical variables. No imputation was made for missing data. The primary outcomes studied were overall survival and incidence of long-COVID-19. The secondary outcomes included changes in laboratory parameters. To determine the independent risk factors for survival, we performed multivariable Cox proportional hazards analysis. Variables were selected for inclusion in the models based on clinical plausibility, statistical significance in the univariable model, and availability in more than 90% of the patients. Multivariable analysis was performed by using Cox proportional hazards analysis.

We compared long-term clinical outcomes of patients with CLD who had COVID-19 and patients with CLD who tested negative for SARS-CoV-2 during the same study period. We used propensity score matching to identify a cohort of patients with CLD without COVID-19 who were statistically matched on a 1:2 basis using the nearest neighbor approach. We matched those with CLD with and without a history of COVID-19 for crucial variables including those known to impact clinical outcomes with COVID-19 including age, sex, race, ethnicity, diabetes mellitus, cardiovascular disease, and cirrhosis. Analyses were performed in SPSS (IBM, USA). Two-sided *p* values were used and considered statistically significant if *p*≤0.05.

## RESULTS

Our study included 807 patients from 17 centers across 8 states and the District of Columbia. After excluding patients who died within 30 days of COVID-19 diagnosis (n=82) and those who had <30 days of follow-up (n=59), 666 patients were included in the final analysis (Supplemental Fig. 1, http://links.lww.com/HC9/A0). The median follow-up of the study was 384 days (range: 31–462 d); 85% were followed for more than 6 months and 58% for more than a year. Over half of the cohort were male (52.8%) with a median age of 58 years (IQR: 47–67). NAFLD was the most common etiology of CLD (50.9%) (Table 1). Chronic hepatitis C was the second common etiology (23.9%), with a majority of these patients having aviremic HCV (59.7%) having attained sustained virologic response. Most patients had noncirrhotic stage disease (76.3%, n=508) at the time of COVID-19 diagnosis, 17.0% (n=113) had compensated cirrhosis, and 6.8% (n=45) had decompensated cirrhosis (Table 1).

**TABLE 1:** Demographic and clinical characteristics of patients with chronic liver disease with history of COVID-19

		All-cause mortality status (n=666)		
	Total (n=666), n (%)	Alive (n=612), n (%)	Died (n=54), n (%)	*p*
Demographic factors				
Age [median (IQR)]	58.0 (19)	57.4 (20)	67 (15)	**<0.001**
Age ≥65 y	214 (32.1)	203 (28.6)	59 (51.8)	**<0.001**
Gender (male)	352 (52.8)	321 (52.5)	31 (57.4)	0.605
Race/ethnicity				0.562
Non-Hispanic White	205 (30.8)	192 (31.4)	13 (24.1)	
Non-Hispanic Black	228 (34.2)	207 (33.8)	21 (38.9)	
Hispanic	156 (23.4)	143 (23.4)	13 (24.1)	
Non-Hispanic Asian	25 (3.8)	21 (3.4)	4 (7.4)	
Other	27 (4.1)	25 (4.1)	2 (3.7)	
Liver-related factors				
Etiology				**<0.001**
HCV	159 (23.9)	136 (22.2)	23 (42.6)	
HBV	44 (6.6)	42 (6.9)	2 (3.7)	
NAFLD	339 (50.9)	324 (52.9)	15 (27.8)	
ALD	61 (9.2)	52 (8.5)	9 (16.7)	
Other	58 (8.7)	53 (8.7)	5 (9.3)	
Severity of liver disease				**<0.001**
No cirrhosis	508 (76.3)	480 (78.4)	28 (51.9)	
Compensated cirrhosis	113 (17.0)	106 (17.3)	7 (13.0)	
Decompensated cirrhosis	45 (6.8)	26 (4.2)	19 (35.2)	
Liver transplant	43 (6.5)	39 (6.4)	4 (7.4)	0.771
Comorbidities				
Diabetes	290 (43.5)	259 (42.7)	31 (57.4)	**0.045**
Hypertension	389 (58.9)	356 (58.6)	33 (61.1)	0.420
Hyperlipidemia	255 (38.6)	241 (39.7)	14 (25.9)	0.057
Obesity	282 (42.7)	16 (29.6)	266 (43.8)	0.055
Cardiovascular disease	107 (16.1)	95 (15.5)	12 (22.2)	0.244
HIV	21 (3.2)	18 (3.0)	3 (5.6)	0.242
COPD	47 (7.1)	45 (7.4)	2 (3.7)	0.415
Asthma	66 (10.0)	63 (10.4)	3 (5.6)	0.346
Tobacco use^a^	69 (10.7)	60 (10.2)	9 (17.6)	0.101
Alcohol use^a^	70 (11.2)	63 (11.1)	6 (12.8)	0.637
Severity of COVID-19				
Mechanical ventilation	102 (15.3)	85 (13.9))	17 (31.5)	**0.002**

Bold values indicate significant *P* ≥ 0.05.

^a^ Ongoing tobacco or alcohol use at the time of diagnosis of COVID-19.

Data are expressed as the median±interquartile range or number (proportion).

Abbreviations: ALD indicates alcohol-related liver disease; COPD, chronic obstructive pulmonary disease.

### Overall survival of patients with CLD and COVID-19

The overall, long-term all-cause mortality was 8.1% (n=54), and delayed COVID-19-related mortality 30 days after the diagnosis of COVID-19 was 3.6% (n=24). The 90-day and 1-year survival rate of the cohort was 97% and 91%, respectively. The 90-day and 1-year COVID-19 specific survival rate was 98% and 96%. During the follow-up period, 34.2% underwent new hospitalizations. We performed univariable and multivariable analysis to identify the predictors of overall long-term survival in patients with CLD and COVID-19 (Table 2). The multivariable model for overall survival was adjusted for variables statistically significant in the univariable model—age, sex, race, ethnicity, etiology of CLD, cirrhosis, hepatic decompensation, diabetes, obesity, and severe COVID-19. The predictors of overall survival were older age [hazards ratio (HR): 1.41, 95% CI: 1.11–1.78 per 10 y], presence of decompensated cirrhosis at the time of diagnosis of COVID-19 (HR: 6.26, 3.35–11.66), diabetes mellitus (HR: 1.97, 1.10–3.53) and severe COVID-19 requiring mechanical ventilation (HR: 2.15, 1.15–3.99) (Table 2). Keeping in line with these findings, in the subgroup of patients for whom model for end-stage liver disease scores were available (n=350), we found that patients with higher model for end-stage liver disease scores at the time of COVID-19 had higher overall long-term mortality (*p*=0.003, HR: 1.06, 1.02–1.10).

**TABLE 2:** Univariable and multivariable analyses: predictors of overall survival among patients with chronic liver disease and who recovered from COVID-19

	Univariable model for overall survival (n=666)		Multivariable model for overall survival (n=666)	
	OR (95% CI)	*p*	OR (95% CI)	*p*
Demographic factors				
Age (per 10 y)	1.49 (1.21–1.84)	**<0.001**	1.41 (1.11–1.78)	**0.005**
Male	1.19 (0.69–2.05)	0.523	1.00 (0.54–1.88)	0.989
Race/ethnicity				
Non-Hispanic white	1		1	
Non-Hispanic black	1.45 (0.73–2.91)	0.290	0.87 (0.19–3.95)	0.859
Hispanic	1.18 (0.80–1.73)	0.396	1.08 (0.25–4.69)	0.920
Non-Hispanic Asian	1.35 (0.93–1.96)	0.111	1.44 (0.31–6.71)	0.646
Other	1.06 (0.73–1.54)	0.758	3.47 (0.60–20.06)	0.164
Liver-related factors				
Etiology of liver disease				
HCV	1		1	
ALD	0.97 (0.45–2.09)	0.929	1.18 (0.39–3.52)	0.772
NAFLD	0.53 (0.38–0.74)	**0.000**	0.877 (0.21–2.87)	0.696
HBV	0.66 (0.41–1.07)	0.096	0.50 (0.16–1.51)	0.220
Other	0.87 (0.69–1.11)	0.280	0.51 (0.09–2.80)	0.435
Severity of liver disease				
No cirrhosis	1		1	
Compensated cirrhosis	0.84 (0.32–2.17)	0.285	0.52 (0.18–1.51)	0.231
Decompensated cirrhosis	7.32 (4.09–13.10)	**<0.001**	6.26 (3.35–11.66)	**<0.001**
Liver transplant recipient	0.92 (0.33–2.54)	0.873		
Comorbidities				
Diabetes	1.79 (1.05–3.07)	**0.034**	1.97 (1.10–3.53)	**0.022**
Hypertension	1.08 (0.63–1.87)	0.779		
Obesity	0.57 (0.31–0.99)	**0.045**	0.94 (0.49–1.84)	0.875
Cardiovascular disease	1.48 (0.78–2.80)	0.234		
Tobacco use	1.96 (0.95–4.03)	0.067	1.21 (0.46–3.16)	0.694
Alcohol use	1.26 (0.53–0.97)	0.596		
Severity of COVID-19				
Mechanical ventilation	2.78 (1.56–4.93)	**<0.001**	2.15 (1.15–3.99)	**0.016**

Bold values indicate significant *P* ≥ 0.05.

The multivariable model for all-cause mortality was adjusted for age, gender, race/ethnicity, etiology of chronic liver disease, cirrhosis, liver transplant, diabetes, hypertension, obesity, smoking status, and alcohol consumption.

Abbreviations: ALD indicates alcohol-related liver disease; ICU, intensive care unit.

To confirm the robustness of our results identifying predictors of overall survival in patients with CLD and COVID-19, we performed sensitivity analysis looking at an earlier time-point 90-day mortality (Supplemental Table 3, http://links.lww.com/HC9/A0). The 90-day mortality was 2.9% (n=19). The results remained stable; older age, presence of decompensated cirrhosis at the time of diagnosis of COVID-19, diabetes mellitus, and severe COVID-19 requiring mechanical ventilation were also independently associated with higher 90-day mortality (Supplemental Table 3, http://links.lww.com/HC9/A0).

### Propensity score matched analysis of CLD patients with and without COVID-19

To evaluate if long-term mortality and morbidity was higher in patients with CLD who had contracted SARS-CoV-2, we used a control group of patients with CLD who tested negative for SARS-CoV-2, during the same time period (n=1998). We built a propensity score model of patients with CLD with and without a history of COVID-19 by matching them for demographic features and known drivers of mortality—age, sex, race, ethnicity, diabetes mellitus, cardiovascular disease, and presence of cirrhosis (Supplemental Table 3, http://links.lww.com/HC9/A0).

**FIGURE 1:**
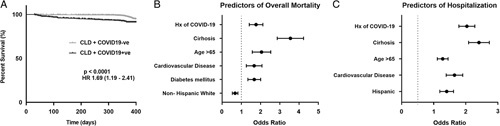
Independent risk for long-term survival and hospitalizations in patients with chronic liver disease (CLD). (A) Overall survival of propensity-matched patients with CLD with (n=666) or without (n=1332) history of coronavirus disease 2019 (COVID-19). (B) Independent predictors of overall mortality in patients with CLD. (C) Independent predictors of hospitalizations in patients with CLD. HR indicates hazards ratio.

After propensity score matching, patients with CLD with history of COVID-19 (n=666) had a higher independent risk for overall mortality (1-year survival 92% vs. 98%, *p*<0.001, HR: 1.69, 1.19–2.41) (Fig. 1A), and hospitalization (34.2% vs. 20.6%, *p*<0.001, HR: 2.0, 1.62–2.48) than patients with CLD without COVID-19 (n=1332), during the follow-up period. Other independent predictors of overall survival were presence of cirrhosis (HR: 3.43, 2.43–4.79), age 65 years or above (HR: 2.04, 1.22–2.88), diabetes mellitus (HR: 1.60, 1.13–2.28), and history of cardiovascular disease (HR: 1.52, 1.04–2.43) (Fig. 1B). Similarly, older age (HR: 1.26, 1.00–1.58), cirrhosis (HR: 2.37, 1.87–2.99), and cardiovascular disease (HR: 1.61, 1.22–2.11) were associated with hospitalizations, along with increased independent risk in Hispanic patients (HR: 1.37, 1.05–1.79) (Fig. 1C).

On subgroup analysis of the adjusted multivariable model, patients who had severe COVID-19 requiring mechanical ventilation had worse overall survival both in those with cirrhosis (*p*<0.001, HR: 2.59, 1.29–5.19) and those without cirrhosis (*p*<0.001, HR: 7.23, 3.62–11.27), compared to CLD without COVID-19. The odds ratio does appear higher in patients without cirrhosis, which we interpret cautiously since the CI is wider given the smaller number of deaths in patients without cirrhosis than in those with cirrhosis. It is possible that severe COVID-19 had a disproportionate impact on patients without cirrhosis than patients with cirrhosis, who already have a higher mortality both with and without COVID-19. In addition, patients with mild/moderate COVID-19 not requiring mechanical ventilation did not have a higher risk for mortality compared to patients with CLD without COVID-19 (*p*=0.09, HR: 1.62, 0.82–2.93), both in patients with and without cirrhosis (Supplemental Figs. 2–3, http://links.lww.com/HC9/A0).

### Resolution of liver injury after COVID-19 in patients with CLD

We evaluated all available follow-up laboratory values for 83% (n=550) of the patients. The following laboratory studies showed significant improvement after resolution of COVID-19: aspartate aminotransferase [median (IQR) peak–>recovery, 58 (65)–>27 (21) IU/ml; *p*<0.0001), ALT [39 (58)–>26 (24) IU/ml; *p*<0.0001], bilirubin [0.6 (0.4)–>0.5 (0.4) mg/dl; *p*<0.0001), albumin [3.0 (1.1)–>3.9 (1.0) g/dl; *p*<0.0001], international normalized ratio [1.2 (0.3)–>1.1 (0.3); *p*<0.0001], and creatinine [1.1 (1.0)–>0.9 (0.5) mg/dl; *p*=0.0002]. In patients who experienced acute liver injury during COVID-19 (n=140), the median ALT decreased from 118 (IQR: 124) during COVID-19 to 34 (IQR: 43) (*p*<0.0001); and median aspartate aminotransferase decreased from 142 (IQR: 141) to 30 (IQR: 33) during follow-up (*p*<0.0001).

**FIGURE 2:**
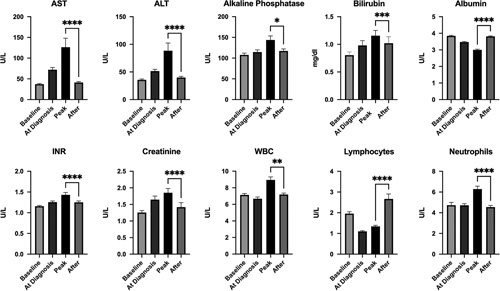
Changes in laboratory parameters in patients with chronic liver disease who recovered from coronavirus disease 2019. ALT, alanine transaminase; AST, aspartate aminotransferase; INR, international normalized ratio; WBC, white blood cells. *p<0.05, **p<0.01, ***p<0.001, ****p<0.0001.

We also observed improvement in peripheral blood cell counts, including leukocyte count [7.3 (5.5)–>6.6 (3.1) million cells/ml; *p*=0.001], lymphocyte count [1.1 (1.0)–>1.8 (1.3 million/ml; *p*<0.0001], and neutrophil count [4.9 (4.8)–>4.1 (2.4) million/ml; *p*<0.0001] (Fig. 2).

### Incidence and predictors of long-COVID-19 in patients with CLD

Overall, 29.9% (n=199) of patients with CLD reported symptoms suggestive of long-COVID-19. The most common symptoms of long-COVID-19 included persistent dyspnea (12.6%), severe fatigue (12.3%), cough (12.2%), arthralgias (8.0%), depression (5.4%), chest pain (5.1%), persistent anosmia (2.6%), and palpitations (1.5%). On multivariable analysis, female sex (HR: 1.45, 1.01–2.11, *p*=0.05), Hispanic ethnicity (HR: 1.94, 1.26–2.99, *p*=0.003), and severe COVID-19 requiring mechanical ventilation (HR: 1.74, 1.06–2.86, *p*=0.028) predicted the occurrence of long-COVID-19. Diabetes and hypertension did not predict long-COVID-19, but obesity was associated with a reduced risk of long-COVID-19 in patients with CLD (HR: 0.45, 0.30–0.66, *p*=0.001). Interestingly, etiology, or severity of CLD were not associated with symptoms of long-COVID-19 (Table 3).

**TABLE 3:** Univariable and multivariable analyses: risk of long-COVID-19 among patients with chronic liver disease with history of COVID-19

	Univariate model for long-COVID-19 (n=629)		Multivariate model for long-COVID-19 (n=629)	
	OR (95% CI)	*p*	OR (95% CI)	*p*
Demographic factors				
Age (>65 y)	0.94 (0.65–1.35)	0.783	0.98 (0.65–1.48)	0.940
Sex (female)	1.24 (0.88–1.74)	0.122	1.45 (1.01–2.11)	**0.050**
Race (White)	0.63 (0.44–0.89)	0.010	0.69 (0.47–1.00)	0.062
Ethnicity (Hispanic)	2.20 (1.49–3.22)	**<0.001**	1.94 (1.26–2.99)	**0.003**
Liver-related factors				
Etiology of liver disease				
HCV	1			
ALD	1.51 (0.80–2.89)	0.240		
NAFLD	0.84 (0.55–1.28)	0.447		
HBV	2.02 (1.01–4.00)	0.059		
Other	1.01 (0.52–1.98)	1.000		
Severity of liver disease				
No cirrhosis	1			
Compensated cirrhosis	1.25 (0.79–1.97)	0.346		
Decompensated cirrhosis	3.82 (2.11–6.91)	0.407		
Liver transplant	0.77 (0.36–1.66)	0.590		
Comorbidities				
Diabetes	0.74 (0.53–1.05)	0.108	0.79 (0.54–1.18)	0.257
Hypertension	0.62 (0.44–0.86)	**0.007**	0.71 (0.48–1.05)	0.090
Tobacco smoker	0.87 (0.49–1.55)	0.775		
Obesity	0.46 (0.32–0.66)	**0.001**	0.45 (0.30–0.66)	**0.001**
Alcohol use	1.30 (0.77–2.21)	0.332		
Severity of COVID-19				
Mechanical ventilation	1.66 (1.06–2.66)	**0.029**	1.74 (1.06–2.86)	**0.028**

Bold values indicate significant *P* ≥ 0.05.

The multivariate model for all-cause mortality was adjusted for age, gender, race/ethnicity, etiology of chronic liver disease, cirrhosis, diabetes, hypertension, smoking status, alcohol consumption and severity of COVID-19.

Abbreviations: ALD indicates alcohol-related liver disease; ALT, alanine transaminase; COPD, chronic obstructive pulmonary disease.

### Vaccine status, lifestyle, and behavioral changes after recovery from COVID-19

COVID-19 vaccine status was available for 72% (n=470) of patients with CLD and history of COVID-19. In total, 70% (n=326) had received the COVID-19 vaccine and 75% (n=243) of the vaccinated patients had received 2 doses of the vaccine (2-dose schedule) at the time of our study completion. Patients of Hispanic ethnicity had a lower vaccination rate than non-Hispanic (59.3% vs. 70.7%; *p*=0.023).

Lastly, we evaluated how COVID-19 impacted the clinical course and behavioral factors in patients with CLD. Despite having a diagnosis of CLD, 33.3% (n=213) of patients were actively drinking alcohol prior to the diagnosis of COVID-19. After recovery from COVID-19, 30.7% (n=192) continued to report active moderate to heavy alcohol use, with 4% (n=25) reporting worse alcohol use and 1.7% (n=11) reporting new alcohol use after recovery from COVID-19. Weight gain was noted in 23% (n=154) of patients, with a median weight gain of 10 pounds (IQR: 14 pounds). Overall, 15% (n=97) experienced disruption in medical care during the follow-up period with clinic visits to hepatologists (10%), imaging appointments (7%), and upper endoscopies (5%) being the most common missed appointments.

## DISCUSSION

In this large, multicenter, longitudinal cohort of patients with CLD and COVID-19, we describe the long-term clinical outcomes and post-COVID-19 sequelae in patients with CLD. We show that patients who survived beyond the acute phase of the illness had a higher risk of long-term mortality and hospitalizations than a matched control group of patients with CLD without COVID-19. Long-COVID-19 was common in patients with CLD, with 29.9% of patients reporting persistent symptoms consistent with long-COVID-19. Severe COVID-19, Hispanic ethnicity, and female sex, but not etiology of CLD or presence of cirrhosis, predicted the development of long-COVID-19. Elevated aminotransferases have been widely reported during COVID-19;^16^–^18^ we now demonstrate that liver injury resolves upon recovery from COVID-19. Thus, our results highlight the prolonged implications of COVID-19 both for recovering patients and for health care systems.

We demonstrate that patients with CLD who contracted COVID-19 had a significantly higher risk of mortality and hospitalization compared to a matched control group of patients with CLD who tested negative for SARS-CoV-2 during the same study period. A few recent studies have demonstrated that COVID-19 is associated with longer-term mortality,^3^,^19^–^21^ however, these studies have not included patients with CLD. Most studies on patients with CLD, including our earlier ones, have only reported on short-term outcomes. The current study now demonstrates the impact of COVID-19 on long-term survival. The higher long-term mortality was significantly higher in those who suffered from severe COVID-19 requiring mechanical ventilation, suggesting this was, at least, partly driven by delayed COVID-19 mortality. Moreover, patients who had decompensated cirrhosis at the time of diagnosis of COVID-19 had a higher independent risk for worse long-term outcomes, potentially due to the negative impact of systemic inflammation induced by SARS-CoV-2 on the more fragile physiological reserve in these patients.^22^,^23^ The mounting evidence that contracting COVID-19 increases the risk of mortality and morbidity even after surviving the acute episode, underlines the critical role of vaccination and mitigation measures to prevent COVID-19.

Long-COVID-19 refers to a wide range of lingering symptoms experienced by patients who have recovered from the acute phase of COVID-19. The prevalence of long-COVID-19 has ranged from 4.7% to 80%, due to variations in its definition.^24^,^25^ Using the WHO definition,^14^ we find that 30% of patients with CLD experienced long-COVID-19. History of severe COVID-19 requiring hospitalization predicted the occurrence of long-COVID-19, while the etiology or severity of liver disease did not pose a risk for long-COVID-19, indicating that these symptoms are likely a consequence of the systemic changes induced by SARS-CoV-2. We conjecture that the impact of severity of cirrhosis on long-COVID may be underestimated in this cohort since patients with decompensated cirrhosis had significantly high mortality during this early phase of COVID-19. Female sex was associated with higher risk for long-COVID-19, consistent with published studies from other populations.^26^,^27^ Moreover, patients of Hispanic ethnicity showed a higher prevalence of severe COVID-19, which is not surprising given the known risk for severe COVID-19 in this cohort.^8^ We have previously shown that socioeconomic factors contribute to the higher risk of COVID-19 in ethnic minorities with CLD.^10^ The continued impact of COVID-19 in this vulnerable population emphasizes the need to evaluate and fill potential gaps in access to health care. Further, the results of our study provide the framework and rationale to pursue further prospective studies to systematically assess the prevalence and pattern of long-COVID-19 in patients with CLD.

Despite carrying a diagnosis of CLD, around a third of patients reported moderate to heavy alcohol use after recovery from COVID-19. This is consistent with published data that have demonstrated increases in alcohol use and alcohol-associated hospitalizations among patients with CLD during the COVID-19 era.^28^–^30^ Prior to the COVID-19 pandemic, the burden of alcohol use disorder and alcohol-related liver disease were on the rise.^31^ With increasing alcohol use and alcohol-related hospitalizations during the pandemic, we suspect that the collateral damage from health-related behavioral factors on patients with CLD is yet to be fully understood.

Furthermore, almost a fourth of patients noted weight gain during the follow-up period, which is in concordance with other recent published work.^32^,^33^ Even prior to the COVID-19 pandemic, the prevalence of both obesity and severe obesity have been increasing among adults living in the US.^34^ Given the trends related to weight gain in patients with CLD during COVID-19, ongoing public health efforts are needed to address the underpinnings for this trend.

Among patients with CLD and a history of COVID-19 in our study, 70% had received at least 1 dose of the COVID-19 vaccine and 75% of those vaccinated had received 2 doses of the vaccine. This is consistent with vaccination rates for the general US population—as of September 2022, 11% of those living in the US were partly vaccinated against COVID-19 and 68% were fully vaccinated against COVID-19.^24^ The data on benefits of COVID-19 vaccination on mortality and severity is well established, both in the general population and in patients with cirrhosis.^35^,^36^ The goal of our study was not to assess the effect of vaccination on mortality or long-COVID-19; however, our results further highlight the importance of continued public health endeavors and individualized counseling to increase uptake of the COVID-19 vaccine, especially among vulnerable subgroups like those with cirrhosis and patients of racial and ethnic minorities, who have lower vaccination rates.^37^

The study has several strengths, including the large sample size, broad geographical distribution across the US, ethnically diverse cohort, and longitudinal follow-up data. Our study results are generalizable given patients treated as outpatients or inpatients as well as patients with noncirrhotic or cirrhotic liver disease were included. Nevertheless, our study should be interpreted with a few limitations in mind. The symptoms of long-COVID-19 were collected by chart review, instead of more direct methods, such as telephone interviews, which may have led to an underestimation of long-COVID-19. However, we used the standard WHO definition of long-COVID-19 and were able to perform a detailed review to assess for alternate explanations for the symptoms, which may not be possible with telephone and internet surveys. The control group was only derived from a single center. However, the availability of a large PCR-confirmed COVID-19 negative cohort is a strength, and we did carefully match the patients for key variables. The patients included in this study were infected in the first wave of the pandemic and the pandemic has evolved since then. Given that millions of patients were affected by COVID-19 during the early phases of the pandemic, our findings remain relevant for patients with CLD who have recovered from COVID-19. Lastly, most institutions included in this cohort are tertiary medical health systems, potentially introducing referral bias.

Our large, multicenter study of long-term follow-up of patients with CLD and COVID-19 provides insight into the natural history of COVID-19 sequelae in patients with CLD. We found a high burden of mortality, morbidity, and long-COVID-19 in patients with CLD. Our data reassuringly demonstrate consistent improvement in laboratory parameters and resolution of liver injury in those who recovered from COVID-19. A confluence of factors including persistent symptoms of COVID-19, ongoing alcohol use, weight gain, and missed medical appointments highlight the need for the medical community to develop strategies to provide ongoing multidisciplinary care for patients with CLD who have recovered from COVID-19.

## Supplementary Material
