# Supplementary material for: Long-term clinical outcomes of patients with COVID-19 and chronic liver disease: US multicenter COLD study
Source: Hepatol Commun. 2023 Jan 3;7(1):e8874. doi: 10.1097/01.HC9.0000897224.68874.de (PMC9827967; doi:10.1097/01.HC9.0000897224.68874.de)
Supplement: Supplementary file 1 [file hc9-7-e8874-s001.docx]

**SUPPLEMENTARY FIGURES AND TABLES**

**Supplementary Figure 1 Study Inclusion and Exclusion Criteria**

**
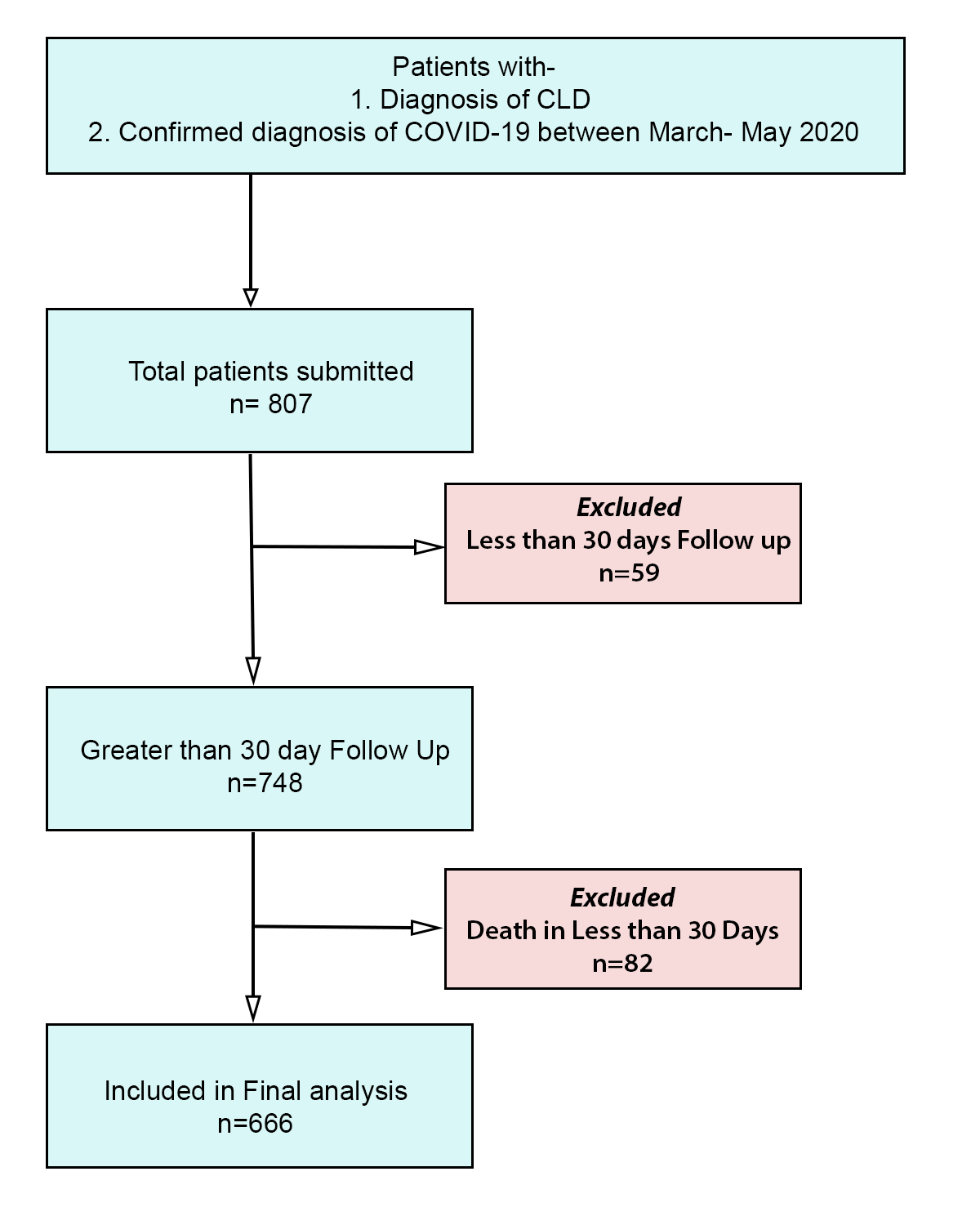
**

Abbreviations: CLD, chronic liver disease; COVID-19, coronavirus disease 2019

**Supplementary Figure 2: All-cause mortality hazard ratios by COVID-19 status and severity**

1. Comparing hazard ratio for all-cause mortality between patients with severe COVID-19 requiring mechanical ventilation and patients with CLD without COVID-19
2. Comparing hazard ratio for all-cause mortality between patients with mild COVID-19 not requiring mechanical ventilation and patients with CLD without COVID-19
3.
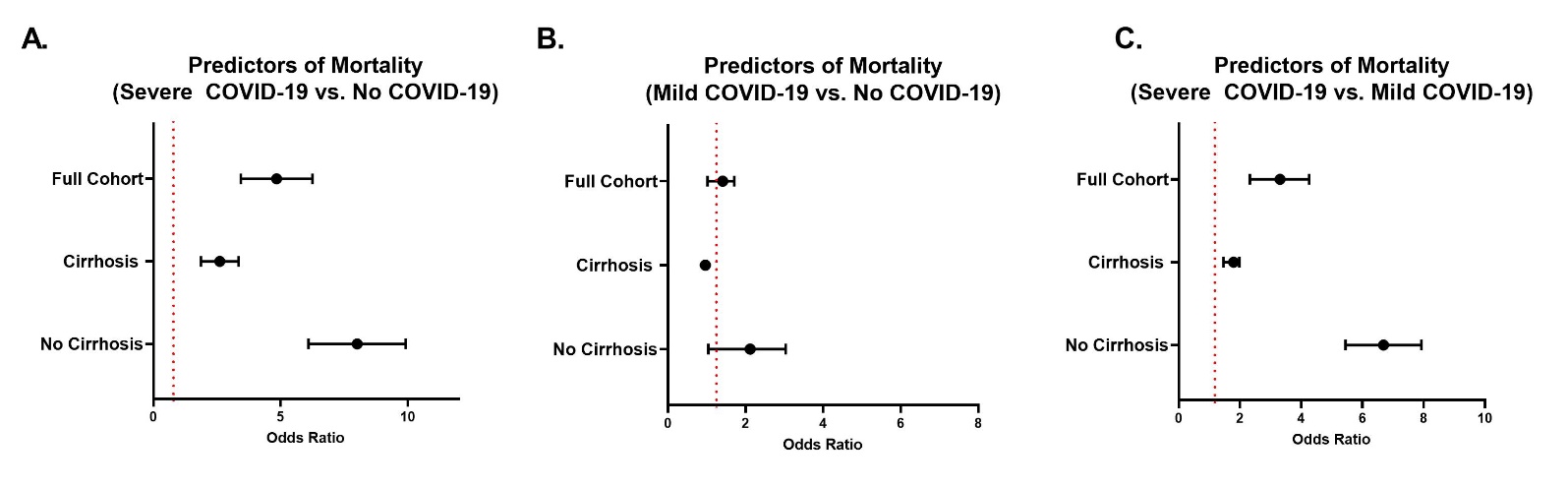
Comparing hazard ratio for all-cause mortality in patients with CLD between those with severe and mild COVID-19.

*The multivariable model for all-cause mortality was adjusted for age, gender, race/ethnicity, etiology of chronic liver disease, diabetes and cardiovascular disease.

**Supplementary Figure 3 Overall Survival by COVID-19 status and severity**


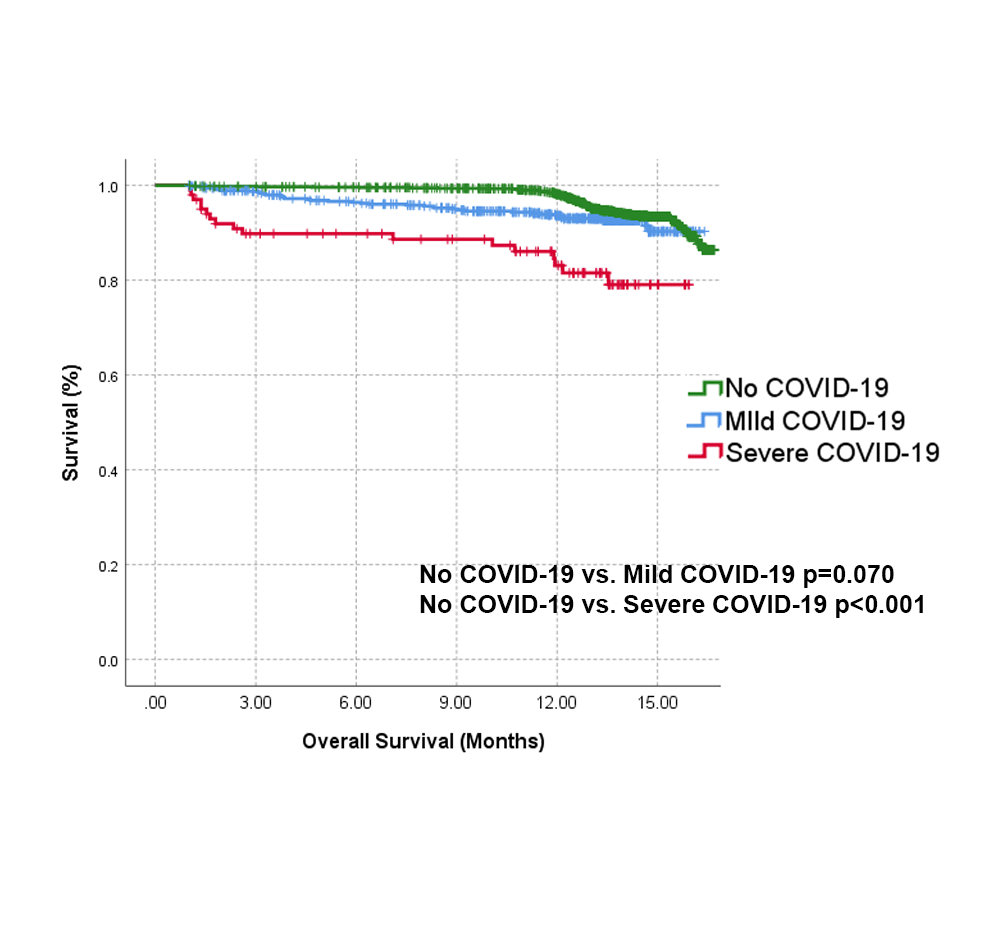


**Supplementary Table 1. List of Participating Institutions**

|  | **Institution** |
| --- | --- |
| 1 | Ochsner Medical Center, LA |
| 2 | Massachusetts General Hospital, MA |
| 3 | University of California San Francisco, Fresno, CA |
| 4 | Hennepin County Medical Center, MN |
| 5 | Beth Israel Deaconess Medical Center, MA |
| 6 | Stanford University, CA |
| 7 | University of Pennsylvania, PA |
| 8 | University of Michigan, MI |
| 9 | Veterans Administration (VA) Medical Center, Washington, DC |
| 10 | University of Minnesota, MN |
| 11 | University of Arizona/Banner Health, AZ |
| 12 | University Hospitals Cleveland Medical Center, OH |
| 13 | University of Southern California, CA |
| 14 | Mayo Clinic, AZ |
| 15 | University of Pittsburgh, PA |
| 16 | Weill Cornell University, NY |
| 17 | Duke University, NC |

**Supplementary Table 2. List of ICD-10 Codes for Chronic Liver Disease**

| **NASH / NAFLD** | **Unspecified chronic liver disease** |
| --- | --- |
| K75.81 Nonalcoholic steatohepatitis (NASH) | K73 Chronic hepatitis |
| K76.0 NAFLD (nonalcoholic fatty liver disease) | K73.0 Chronic persistent hepatitis |
| **Alcohol-related liver disease** | K73.1 Chronic lobular hepatitis |
| K70 Alcoholic liver disease | K73.2 Chronic active hepatitis |
| K70.1 Alcoholic hepatitis | K73.8 Other chronic hepatitis, Recurrent hepatitis |
| K70.10 …… without ascites | K73.9 Chronic hepatitis, unspecified |
| K70.11 …… with ascites | K74 Fibrosis and cirrhosis of liver |
| K70.2 Alcoholic fibrosis and sclerosis of liver | K74.0 Hepatic fibrosis |
| K70.3 Alcoholic cirrhosis of liver | K74.1 Hepatic sclerosis |
| K70.30 …… without ascites | K74.2 Hepatic fibrosis with hepatic sclerosis |
| K70.31 …… with ascites | K74.4 Secondary biliary cirrhosis |
| K70.4 Alcoholic hepatic failure | K74.5 Biliary cirrhosis, unspecified |
| K70.40 …… without coma | K74.6 Other and unspecified cirrhosis of liver |
| K70.41 …… with coma | K74.60 Unspecified cirrhosis of liver |
| K70.9 Alcoholic liver disease, unspecified | K74.69 Other cirrhosis of liver |
| **Chronic Hep C/Hep B** | K71.7 Toxic liver disease with fibrosis and cirrhosis |
| B18.2 Chronic hepatitis C | K71.3 Toxic liver disease with chronic hepatitis |
| K74.6 Chronic hepatitis C with cirrhosis | K71.4 Toxic liver disease with chronic lobular hepatitis |
| K74.69, B19.2 cirrhosis to HCV | K71.5 Toxic liver disease with chronic active hepatitis |
| B18.1 Chronic hepatitis B | K71.50 …… without ascites |
| K74.6, B19.1 Chronic hepatitis B with cirrhosis | K71.51 …… with ascites |
| **PBC/PSC/Autoimmune hepatitis** | K76.6 Portal hypertension |
| K74.3 Primary biliary cirrhosis | K76.7 Hepatorenal syndrome |
| K74.3 Cirrhosis due to primary sclerosing cholangitis | K76.81 Hepatopulmonary syndrome |
| K83.01 Primary sclerosing cholangitis | **Decompensated cirrhosis** |
| K75.4 Autoimmune hepatitis | K72.9 Decompensated hepatic cirrhosis |
|  | K74.69 Decompensated liver disease |

**Supp Table 3. Univariable and Multivariable Analyses: Risk of Short-Term Mortality (90-day) among Patients with Chronic Liver Disease and who Recovered from COVID-19**

|  | Univariable model for 90-day mortality (n=666) | | Multivariable model for 90-day mortality (n=666) | |
| --- | --- | --- | --- | --- |
|  | OR (95% CI) | *P* value | OR (95% CI) | *P* value |
| Demographic factors |  |  |  |  |
| Age (>65 years) | 1.49 (1.21-1.84) | **<0.001** | 2.90 (1.01-8.34) | **0.048** |
| Male | 1.19 (0.69-2.05) | 0.523 | 1.26 (1.13-1.53) | 0.265 |
| Race/ethnicity |  |  |  |  |
| Non-Hispanic white | 1 |  | 1 |  |
| Non-Hispanic black | 1.11 (0.60-2.04) | 0.791 | 1.13 (0.11-11.55) | 0.916 |
| Hispanic | 0.85 (0.53-1.36) | 0.737 | 1.13 (0.11-11.11) | 0.911 |
| Non-Hispanic Asian | 1.04 (0.77-1.41) | 0.558 | 1.58 (0.15-18.79) | 0.713 |
| Other | 1.03 (0.76-1.41) | 0.584 | 1.96 (0.07-49.54) | 0.683 |
| Liver-related factors |  |  |  |  |
| Etiology of liver disease |  |  |  |  |
| HCV | 1 |  |  |  |
| ALD | 1.08 (0.68-1.74) | 0.711 |  |  |
| NAFLD | 0.68 (0.38-1.24) | 0.365 |  |  |
| HBV | 0.78 (0.72-0.84) | 0.344 |  |  |
| Other | 1.10 (0.69-1.76) | 0.703 |  |  |
| Severity of Liver Disease |  |  |  |  |
| No Cirrhosis | 1 |  | 1 |  |
| Compensated cirrhosis | 0.99 (0.78-1.28) | 1.000 | 1.91 (0.35-10.47) | 0.457 |
| Decompensated cirrhosis | 1.59 (1.06-2.35) | **<0.001** | 14.79 (2.41-60.58) | **0.004** |
| Liver Transplant Recipient | 1.11 (0.92-1.36) | 0.118 |  |  |
| Comorbidities |  |  |  |  |
| Diabetes | 3.63 (1.28-10.27) | **0.034** | 8.18 (2.05-32.72) | **0.008** |
| Hypertension | 1.58 (0.74-3.37) | 0.239 |  |  |
| Obesity | 0.90 (0.64-1.28) | 0.647 |  |  |
| Cardiovascular Disease | 0.94 (0.85-1.05) | 0.710 |  |  |
| Tobacco Use | 1.06 (0.87-1.29) | 0.446 |  |  |
| Alcohol Use | 0.88 (0.86-0.91) | 0.241 |  |  |
| Severity of COVID-19 |  |  |  |  |
| Mechanical Ventilation | 1.67 (1.09-2.56) | **<0.001** | 6.94 (2.41-20.05) | **<0.001** |
| ICU for COVID-19 | 8.86 (1.32-59.85) | **<0.001** |  |  |
| Vasopressor Use | 1.71 (1.12 -2.63) | **<0.001** |  |  |

Abbreviations: COVID-19, coronavirus disease 2019; OR, odds ratio; CI, confidence interval; HCV, hepatitis C virus infection; HBV, hepatitis B virus infection; NAFLD, nonalcoholic fatty liver disease; ALD, alcohol related liver disease; ICU Intensive Care Unit

The multivariable model for all-cause mortality was adjusted for age, gender, race/ethnicity, etiology of chronic liver disease, cirrhosis, liver transplant, diabetes, hypertension, obesity, smoking status, and alcohol consumption.

**Supplementary Table 4. Comparison of matched and unmatched CLD+COVID-19+ve and CLD+COVID-19-ve cohorts**

| **Category** | **Variable** | **COLD Study**  **CLD+COVID-19+**  **(n=666)** | **Unmatched**  **CLD+COVID-19-**  **(n=1998)** | **p value** | **Matched**  **CLD+COVID-19-**  **(n=1332)** | **p value** |
| --- | --- | --- | --- | --- | --- | --- |
| Demographics | Age>65 | 214 (32.1) | 781 (39.1%) | **<0.0001** | 383 (29.5) | 0.236 |
|  | Male | 352 (52.9) | 1024 (51.3) | 0.502 | 724 (54.4) | 0.536 |
|  | Race (NHW) | 205 (30.8) | 865 (43.3) | **0.002** | 418 (31.4) | 0.798 |
|  | Ethnicity (Hispanic) | 156 (23.4) | 439 (22.0) | 0.761 | 323 (24.2) | 0.698 |
| Comorbidities | DM | 290 (43.5) | 587 (29.4) | **<0.0001** | 511 (38.4) | 0.059 |
|  | CVD | 107 (16.1) | 310 (15.5) | 0.758 | 213 (16.0%) | 1.000 |
| Cirrhosis | Yes | 158 (23.7) | 333 (16.7) | **<0.0001** | 277 (20.8%) | 0.135 |

Abbreviations: CLD, chronic liver disease; COVID 2019, coronavirus disease-19; DM, diabetes; NHW, non-Hispanic white, CVD- cardiovascular disease.
